# Tumescentless endovenous radiofrequency ablation with local hypothermia and compression technique

**DOI:** 10.5830/CVJA-2013-053

**Published:** 2013-10

**Authors:** Kemal Korkmaz, Ali Ümit Yener, Hikmet Selçuk Gedik, Ali Baran Budak, Serhat Bahadir Genç, Özlem Yener, Ayşe Lafçi

**Affiliations:** Department of Cardiovascular Surgery, Numune Research and Training Hospital, Ankara, Turkey; Department of Cardiovascular Surgery, Numune Research and Training Hospital, Ankara, Turkey; Department of Cardiovascular Surgery, Numune Research and Training Hospital, Ankara, Turkey; Department of Cardiovascular Surgery, Numune Research and Training Hospital, Ankara, Turkey; Department of Cardiovascular Surgery, Numune Research and Training Hospital, Ankara, Turkey; Department of Radiology, Ankara Yuksek Ihtisas Research and Training Hospital, Ankara, Turkey; Department of Anaesthesiology, Numune Research and Training Hospital, Ankara, Turkey

**Keywords:** radiofrequency ablation, tumescentless, insufficiency of great saphenous vein

## Abstract

**Introduction:**

Modern surgical management of chronic venous insufficiency is possible since the development of catheter-based minimally invasive techniques, including radiofrequency ablation (RFA) and the application of colour Doppler sonography. RFA technology requires the use of tumescent anaesthesia, which prolongs the operating time. Instilling tumescent anaesthesia percutaneously below the saphenous fascia is the steepest part of the learning curve. In our study, we compared operative and postoperative results of tumescentless RFA and RFA with tumescent anaesthesia, to investigate the necessity of tumescent anaesthesia.

**Methods:**

A total of 344 patients with Doppler-confirmed great saphenous vein insufficiency underwent RFA between January and December 2012. Patients were divided into two groups according to anaesthetic management. Group 1 consisted of 172 patients: tumescent anaesthesia was given before the ablation procedure, and group 2 contained 172 patients: a local hypothermia and compression technique was used; no tumescent anaesthesia was administered. The visual analogue scale (VAS) was used and ecchymosis scores of the patients were recorded. Clinical examinations were performed at each visit and Doppler ultrasonography was performed in the first and sixth month.

**Results:**

Mean ablation time was significantly lower in group 2 compared to group 1 (7.2 vs 18.9 min; *p* < 0.05). Skin burn and paresthesia did not occur. The immediate occlusion rate was 100% for both groups. No significant difference was found between the groups in terms of VAS and ecchymosis scores. All patients returned to normal activity within two days. The primary closure rate of group 1 was 98.2% and group 2 was 98.8% at six months, and there was no significant difference between the groups (*p* > 0.05).

**Conclusion:**

Eliminating tumescent infusion is a desirable goal. Tumescentless endovenous RFA with local hypothermia and compression technique appears to be safe and efficacious. Our technique shortens the operation time and prevents patient procedural discomfort.

## Abstract

Chronic venous insufficiency (CVI) is a frequent disease affecting approximately 20 to 40% of people in Western society.^1^ Generally, venous reflux at the great saphenous vein (GSV) and/or saphenofemoral junction (SFJ) is the commonest cause, leading to varicose veins and associated symptoms such as leg pain, itching, fatigue, night cramps and a burning sensation.^2^,^3^ In severe cases, swelling, skin changes and venous ulceration may develop. CVI has also been related to thrombophlebitis and pulmonary embolism.^4^

The GSV ligation and stripping (L/S) operation was the only treatment of choice from 1950 until recently.^5^ However, it is invasive, has a recurrence rate of approximately 30%, and is not conservative.^3^,^6^ Furthermore, Wood *et al.* reported the rate of cutaneous nerve injury as 27% after L/S.^7^

Modern surgical management of insufficiency of the GSV is possible because of the development of catheter-based minimally invasive techniques, including radiofrequency ablation (RFA) and the application of colour Doppler sonography (CDS). RFA is an alternative technique to L/S, working by ablating the vein using thermal energy delivered through an RF catheter which is inserted into the target vein under CDS guidence. It has been recognised that RFA reduces postoperative recovery time, postoperative pain, wound-related complications, and enables earlier return to normal activities.^8^-^10^

RFA technology requires the use of tumescent anaesthesia before the delivery of thermal energy through the catheter. Tumescent anaesthesia provides a heat sink to prevent the radiation of thermal energy to the surrounding tissues and increases contact between the RF catheter and the vein wall by mechanically reducing the luminal diameter.^11^,^12^ However, it prolongs the operation time and can be a source of patient procedural discomfort. Furthermore instilling tumescent anaesthesia percutaneously below the saphenous fascia under CDS guidance is the steepest part of the learning curve. In our study, we compared the operative and postoperative results of tumescentless RFA and RFA with tumescent anaesthesia to investigate the necessity of tumescent anaesthesia.

## Methods

Patients underwent a physical examination by a vascular surgeon. All the treated patients were symptomatic. Symptoms included pain, itching, limb heaviness, cramps, restless leg and distress about cosmetic appearance. CEAP (Clinical, aEtiology, Anatomical and Pathology) class was determined. All the patients’ height and body weights were measured. The body mass index was verified with the following formula: weight (kg)/height^2^ (m).^13^

In all patients, the potential risks and benefits of endovenous radiofrequency ablation therapy were explained, and written informed consent was obtained. Additionally, throughout the study the principles of the Helsinki Declaration were strictly followed.

Doppler venous scanning was performed on all patients in order to document the extent and severity of the reflux in the great saphenous vein and to evaluate the deep venous system. Doppler imaging of the patients was performed by Aloka Prosound Alpha 7 (Hitachi Aloka Medical, Japan) using 5- and 7-mHz linear probes.

Reflux was determined at the saphenofemoral and saphenopoliteal junctions in the standing position using the Valsalva manoeuvre or manual distal compression with rapid release. Pathological venous reflux was defined as a reverse flow extending for 0.5 seconds or longer. The localisation and severity of the venous reflux and sonographic distribution of the varicose veins were recorded.

Other parameters measured using grey-scale ultrasound in the standing position are as follows:

• the diameter of the GSV in the SFJ
• the diameter of the GSV above the knee
• the distance of the GSV from the skin above the knee
• the distance of the GSV from the skin in the middle of the thigh
• the length of the GSV that was to be ablated.

Only patients with documented GSV reflux with Doppler ultrasonography and in CEAP class 2 or above were recruited into the study. Patients were excluded if there was a significant reflux in the deep venous system, small saphenous vein or perforators. Other exclusion criteria included: deep-vein thrombosis, superficial thrombophlebitis, peripheral arterial vascular disease, immobility, pregnant or breast-feeding patients, and previous history of allergy to local anaesthesia.

All cases in this study were performed under general anaesthesia using a laryngeal mask combined with either tumescent anaesthesia or a local hypothermia and compression technique. Electrocardiogram, arterial pressure and oxygen saturation of the patients were continously monitored. Induction was achieved with midazolam (0.03 mg/kg), lidocain (1 mg/kg) and propofol (2 mg/kg). Anaesthesia was maintained with 2% sevoflurane.

Between January and December 2012 we treated 344 patients in CEAP clinical class 2–6 with endovenous radiofrequency ablation. These patients were divided into two groups according to the anaesthetic management as follows. Group 1: 58 males, 114 females (*n* = 172). Tumescent anaesthesia was given before the ablation procedure. Group 2: 42 males, 130 females (*n* = 172). The procedure was performed without tumescent anaesthesia. Local hypothermia and compression technique was used.

Patients were placed in the supine position. Prior to performing RFA, Doppler ultrasonography was used to confirm the important parameters, including imaging of the GSV and SFJ, perforators, tributaries, and diameter and treatment length, to devise an effective operative plan.

A linear 5- or 7-MHz probe was inserted into a sterile cover and using ultrasound guidance, the GSV was cannulated below or just above the knee. Following the introduction of a 0.025-inch guidewire into the GSV, a 4- or 5-Fr intraducer sheath was advanced over it. The RFA catheter (ClosureFast radiofrequency ablation catheter, NYSE:COV) was then placed through the sheath, the guidewire was removed and the tip of the catheter was placed 2–3 cm distal to the SFJ, just below the superficial epigastric vein, under ultrasound guidance.

**Fig. 1.**
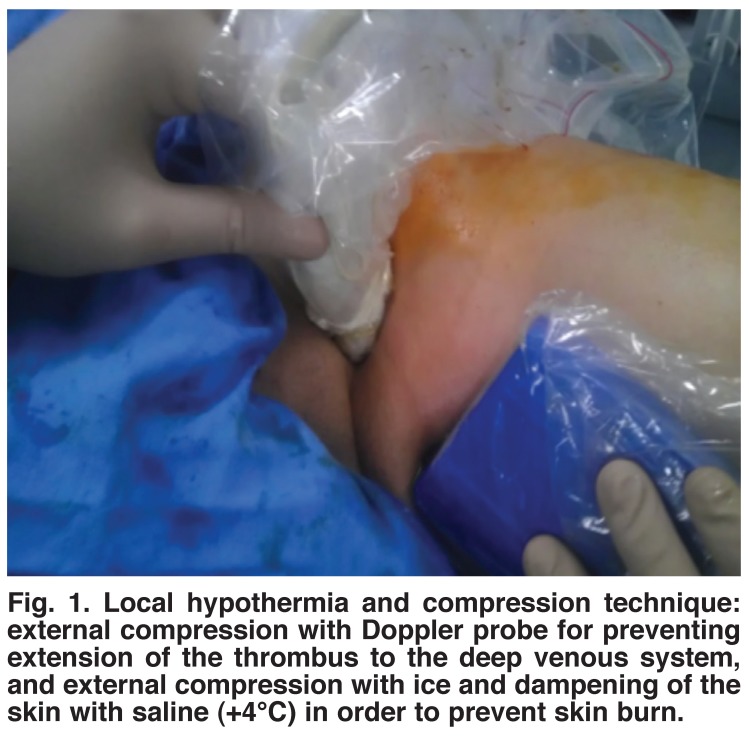
Local hypothermia and compression technique: external compression with Doppler probe for preventing extension of the thrombus to the deep venous system, and external compression with ice and dampening of the skin with saline (+4°C) in order to prevent skin burn.

Once proper positioning was confirmed with ultrasound, a tumescent anaesthetic solution was instilled percutaneously below the saphenous fascia to surround the vein in group 1 patients. This solution consisted of 500 ml saline, 20 ml 2% prilocaine, 20 ml 8.4% sodium bicarbonate and 0.5 ml epinephrine. In group 2, instead of local tumescent anaesthesia, we used a local hypothermia technique (external compression with ice and dampening the skin with saline (+4°C) in order to prevent skin burn) (Fig. 1).

**Fig. 2.**
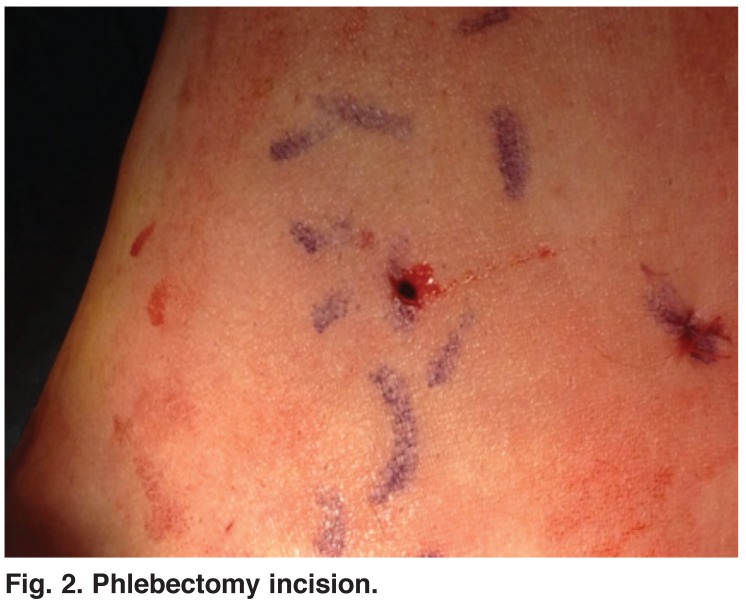
Phlebectomy incision.

**Fig. 3.**
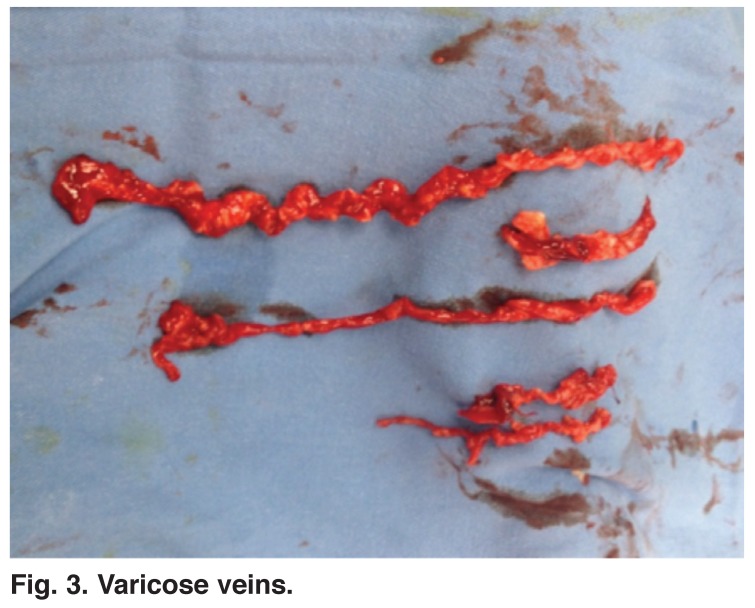
Varicose veins.

The RF generator (VNUS Medical Technologies) was then activated and delivery of radiofrequency energy was maintained at 120°C. Radiofrequency ablation was performed at a rate of 40 W per 7 cm. During the procedure, in both groups, sufficient pressure was exerted with the ultrasound probe to occlude the SFJ and CFV. Following completion of the procedure, closure of the GSV and patency of the common femoral vein and superficial epigastric vein was checked with Doppler ultrasound. As the last step of the treatment, all varicose veins were removed by phlebectomy in both groups (Figs 2, 3).

A compression bandage was wrapped around the treated limb and the patient was encouraged to walk immediately. This remained in place for three hours. The patients wore class II (30–40 mmHg) thigh-high compression stocking continously for the next 24 hours. They then wore the compression stocking only during the day for the next 15 days. The patients were prescribed a non-steroidal anti-inflammatory drug, an antibiotic and a venoactive drug during the postoperative period.

Patients were followed up in the third hour, seventh day, and first and sixth month post-procedure. The patients registered a pain score on a visual analogue scale (VAS) from 0 (no pain) to 10 (worst pain ever). Also the ecchymosis scores of the patients were scaled from 0 (light bruise) to 5 (critical bruise). Clinical examination was performed at each visit and Doppler ultrasonography was performed on the first and sixth months postoperatively. The initial clinical result were assessed, GSV diameters were measured and occlusion of GSV was confirmed with ultrasonography.

## Statistical analysis

Data analysis was performed using the SPSS for Windows 11.5 package program. The Shapiro Wilk test was used in order to investigate whether the distribution of continous variables was close to normal. Measurable parameters were expressed as mean ± standard error of the mean. Intergroup comparisons were made with the Student’s *t*-test or Mann-Whitney *U*-test, as appropiate, for normally and non-normally distributed data, respectively. Nominal variables were evaluated with Pearson’s chi-square test. A *p* value < 0.05 was considered statistically significant.

## Results

A total of 344 patients were treated with endovenous radiofrequency ablation. In group 1, tumescent anaesthesia was given before the ablation procedure while in group 2, a local hypothermia technique was used. The demographic data of the patients are shown in Table 1. There was no significant intergroup differences in terms of age, gender, body weight and height, and body mass index (*p* > 0.05)

**Table 1:** Demographic Data Of The Patients

*Variables*	*Group 1 (n = 172)*	*Group 2 (n = 172)*	*p-value*
Age (years)	46.1 ± 10.2	44.0 ± 10.6	0.178
Gender (f/m)	114/58	130/42	0.179
Height (cm)	167.2 ± 7.6	165.4 ± 8.1	0.146
Weight (kg)	79.0 ± 10.9	76.5 ± 13.4	0.173
Body mass index (kg/m^2^)	28.3 ± 3.8	28.0 ± 4.7	0.592

No statistically significant difference was found between the groups in the pre-operative grey-scale measurements of GSV, including diameter of the GSV in the SFJ, the diameter of the GSV above the knee, the distance of the GSV from the skin above the knee, and the length of the GSV that was to be ablated (*p* > 0.05). However, the distance of the GSV from the skin in the middle of the thigh was shorter in group 1 patients (*p* = 0.017) (Table 2).

**Table 2:** Pre-Operative Grey-Scale Measurements Of The GSV

*Measurement (cm)*	*Group 1 (n = 172)*	*Group 2 (n = 172)*	*p-value*
Diameter of GSV above the knee	5.0 (4.0–12.1)	4.8 (4.0–11.9)	0.532
Diameter of GSV in SFJ	9.2 (6.2–23.0)	9.2 (4.9–23.0)	0.952
Length of GSV treated	44.0 (30.0–70.0)	42.5 (20.0–64.0)	0.147
GSV-skin distance above the knee	8.0 (1.3–28.8)	9.2 (1.0–32.2)	0.099
GSV-skin distance mid-thigh	11.3 (2.6–32.5)	14.0 (1.0–34.7)	0.017

Mean ablation time was significantly lower in group 2 compared to group 1 (7.2 vs 18.9 min; *p* < 0.05). Skin burn and paresthesia did not occur. Immediate occlusion rate was 100% for both groups. All patients returned to normal activity within two days.

All patients have reached the six-month follow-up point. We recognised recanalisation in three patients in group 1 and two in group 2 by Doppler ultrasound scanning. The primary closure rate of group 1 was 98.2% and group 2 was 98.8% at six months and there was no significant difference between the groups (*p* > 0.05). Endovenous heat-induced thrombosis and deep-vein thrombosis were not observed in any of the patients.

The patients’ VAS scores were measured in the third hour, seventh day, first month and sixth month (Table 3). We observed there was no statistically significant difference between the pain score for the patients who recieved tumescent anesthesia and the patients on whom a local hypothermia and compression technique was used (*p* > 0.05).

**Table 3:** Postoperative Visual Analogue Scale Scores Of The Patients

*Postoperative time*	*Group 1*	*Group 2*	p*-value*
3rd hour	2 (0–7)	2 (0–8)	0.231
7th day	0 (0–1)	0 (0–1)	0.189
1st month	0 (0–5)	0.5 (0–6)	0.105
6th month	0 (0–1)	0 (0–1)	1.000

The patients’ ecchymoses scores were measured in the third hour, seventh day, first month and sixth month (Table 4). There was no significant difference between the groups in terms of ecchymoses scores (*p* > 0.05).

**Table 4:** Postoperative Ecchymosis Scores Of The Patients

*Postoperative time*	*Group 1*	*Group 2*	*p-value*
3rd hour	0 (0–2)	0 (0–3)	0.405
7th day	0 (0–0)	0 (0–0)	1.000
1st month	0 (0–0)	0 (0–0)	1.000
6th month	0 (0–0)	0 (0–0)	1.000

At the one-month and six-month Doppler ultrasonography follow up, the diameter of the GSV at the SFJ and above the knee were measured. In both groups, GSV had decreased in diameter but no significant difference was found between the groups (Table 5).

**Table 5:** Diameter Of GSV At SFJ And Above The Knee At One-Month And Six-Month Doppler Ultrasound Follow Up

*Diameter of GSV (cm)*	*Group 1*	*Group 2*	p*-value*
1st month above the knee	4.2 (0.0–10.5)	4.2 (2.7–10.5)	0.753
1st month at SFJ	8.3 (4.7–21.7)	8.4 (4.1–22.1)	0.480
6th month above the knee	3.5 (2.4–8.8)	3.7 (2.2–8.1)	0.960
6th month at SFJ	7.0 (3.8–18)	7.0 (3.8–20.1)	0.971

## Discussion

Until recently, conventional treatment for insufficiency of the GSV was ligation over the saphenous–femoral arch and stripping of the GSV. However this surgical operation has a long postoperative recovery time, a high incidence of postoperative paresthesias, haematoma formation and pain, a high recurrence rate, and is associated with wound-related complications such as infection.^8^,^9^,^14^,^15^ Understanding the pathophysiology of venous insufficiency, and technological advancement have enabled minimally invasive methods to be used for ablation of the GSV.

RF consists of electric and magnetic waves that transform into thermal energy after contact with tissue.^16^ The increased luminal temperature induces vein wall contraction via denaturation of the collagen matrix. Various macro- and microscopic changes occur, including endothelial destruction, shortening and thickening of the venous wall and fibrotic sealing of the vessel lumen.^16^,^17^ Lohr *et al*. pointed out that the temperature gradient between the intima and adventitia and duration of the time of heating determines the total injury to the vein wall collagen and subsequently the total shrinkage of the vein wall.^11^

Earlier studies using first-generation RF devices and catheters^17^,^18^ maintained a set point temperature of 90°C and a slow incremental pull-back catheter technique with a speed of 2–3 cm/min. Merchant et al.^19^ pointed out the importance of the pull-back speed and its effect on the success of the venous closure. They also reported a higher incidence of clot formation, early vessel recanalisation and thermal injury with this technique.19 These issues are not considered to be relevant with the development of the latest generation catheters such as ClosureFast.

Kapoor *et al*. retrospectively analysed the data of 100 patients treated with ClosureFast and showed that the new technique offsets the limitations of previous RF techniques, leads to good venous closure with minimal complications and improves patient comfort.^20^ This was the reason for us using the latest generation catheter in our study.

The ClosureFast catheter treats a 7-cm segment of vein, providing an improved segmental contact with the vessel wall and has a lubricious jacket to ease guidance and decrease the formation of clotting.^17^ There is a temperature sensor near the catheter tip. The RF generator provides transmural heating of the venous wall and limits heating up of the surrounding tissue by using minimum energy (15–40 W) to reach and maintain a temperature set point of 120°C during the 20-s cycles.^11^,^16^

Tumescent anaesthesia isolates the GSV from the surrounding soft tissue and by creating a heat sink, it prevents transfer of the thermal effects of intravascular energy to non-target tissues. Furthermore, it mechanically decreases the luminal diameter of the vein to better contact the laser or RF catheters, and drains the blood from inside the lumen to decrease thrombus formation.^11^,^12^,^21^

Merchant *et al.* showed a reduction in the incidence of paresthesia and rate of skin burn after the use of tumescent infiltration.^22^ However, it increases the operation time, its placement requires multiple patient needle-sticks, causing brusing, and it is the most difficult part of the learning curve.^12^ Besides Gibson *et al.* drew attention to the difficulty of adjusting the correct amount of tumescent solution.23 Technological advances in RF catheters have decreased the risk of pain, bruising and thermal injury to the nerve, muscle or skin.

The aim of our study was elimination of tumescent infusion. Avoiding tumescent anaesthesia in group 2 reduced the ablation time significantly, as expected. Most importantly, however, the primary closure rates were similar at six months and no significant difference was found between the groups in terms of VAS and ecchymosis scores, postoperative measured GSV diameters, and the time needed to return to daily activities.

The great saphenous nerve is very close to the GSV below the knee. We aimed to avoid nerve injury by performing venous puncture above the knee. With the technological advantages of the ClosureFast catheter, we did not encounter paresthesia in the tumescentless group.

Our technique included external compression with ice and dampening of the skin with saline (+4°C), thereby cooling the skin and providing local hypothermia to prevent skin burn. In group 1, we used tumescent anaesthesia. No skin burn was found in either group.

RFA of the GSV with a diameter greater than 12 mm has been another controversial issue, especially for first-generation catheters. Manfrini *et al.* reported that tumescent anaesthesia has a critical role to play in improving venous closure rates by compressing the vein wall into close contact with the catheter.^24^ Merchant *et al.* treated 59 limbs with GSV diameters greater than 12 mm and reported an occlusion rate of 96% at six months.^22^

ClosureFast fits through a 7-Fr sheath, improving the segmental contact with the vessel wall. Proebstle *et al.* treated 252 GSV with ClosureFast with diameters as large as 18 mm.^25^ They used tumescent anaesthesia and the primary venous closure rate was 99.6% at six months. Calcagno *et al.* retrospectively analysed 338 patients and concluded that vein diameter exceeding 12 mm had no effect on closure rate with the ClosureFast catheter, and using tumescent anaesthesia should allow for the succesful treatment of large veins.^26^

We did not find any reports investigating the effect of ClosureFast without tumescent anaesthesia on vein diameters > 12 mm. The mean diameter of GSV was 9.2 mm but the range extended up to 23 mm in both groups. Considering our results, we can claim that tumescentless RFA using a local compression and hypothermia technique was as succesful as RFA using tumescent anaesthesia for GSV diameters exceeding 12 mm.

Deep-vein thrombosis after varicose vein surgery is a wellknown risk.^27^ With the development of special endoluminal catheters, a potential complication of the new techniques was reported: endovenous heat-induced thrombosis (EHIT).^28^ This can briefly be described as a thrombus extending from the superficial venous system to the deep venous system.

In our technique, we externally compress the SFJ with the Doppler probe in order to prevent extension of the thrombus. Marsh *et al.* performed RFA on 2 470 limbs and identified deepvein thrombosis (DVT) in 17 limbs (0.7%).^27^ Of these, four were EHIT. Neither EHIT nor DVT was seen in our study, possibly due to keeping the tip of the catheter 2 cm distal to the SFJ and avoidance of the propagation of thrombus by compression of the SFJ.

## Conclusion

Eliminating tumescent infusion is a desirable goal. Tumescentless endovenous RFA with a local hypothermia and compression technique appears to be safe and efficacious. Our technique shortens the operating time and prevents patient procedural discomfort.
